# Relationship of Rice Grain Amylose, Gelatinization Temperature and Pasting Properties for Breeding Better Eating and Cooking Quality of Rice Varieties

**DOI:** 10.1371/journal.pone.0168483

**Published:** 2016-12-19

**Authors:** Yunlong Pang, Jauhar Ali, Xiaoqian Wang, Neil Johann Franje, Jastin Edrian Revilleza, Jianlong Xu, Zhikang Li

**Affiliations:** 1 Institute of Crop Science/National Key Facility for Crop Gene Resources and Genetic Improvement, Chinese Academy of Agricultural Sciences, Beijing, China; 2 International Rice Research Institute, Metro Manila, Philippines; Institute of Crop Science, CHINA

## Abstract

A total of 787 non-waxy rice lines– 116 hybrids and 671 inbreds–were used to study the apparent amylose content (AAC), gelatinization temperature (GT), and rapid visco analyzer (RVA) pasting viscosity properties of rice starch to understand their importance in breeding better rice varieties. The investigated traits showed a wide range of diversity for both hybrid (HG) and inbred (IG) groups. The combinations of the different categories of AAC and GT were random in HG but were non-random in IG. For inbred lines, the high level of AAC tended to combine with the low level of GT, the intermediate level of AAC tended to have high or intermediate GT, and the low level of AAC tended to have high or low GT. Some stable correlations of the AAC, GT, and RVA properties may be the results derived from the physicochemical relationships among these traits, which rice breeders could utilize for selection in advanced breeding generations. Through cluster analysis, IG and HG were divided into 52 and 31 sub-clusters, respectively. Identifying the cultivars having AAC, GT, and RVA properties similar to that of popular high-quality rice varieties seems to be an interesting strategy and could be directly used for adaptation trials to breed high-quality rice varieties in targeted areas in a more customized manner.

## Introduction

Rice (*Oryza sativa* L.) is one of the most important staple food crops of the world. Each year, a number of new rice varieties are bred and released continuously which have higher yield potentials and better resistance to abiotic and biotic stresses. However, the grain quality of such varieties is often ignored. Currently, with the rapid economic growth and improved living standards in many Asian countries, there is a steady demand for high-quality rice varieties. Breeders should, therefore, develop rice cultivars that have desirable grain quality, customized to meet the market requirements in target regions. In many breeding programs, the improvement of the quality of rice, especially its eating and cooking quality (ECQ), is an important objective as rice is mainly consumed in cooked form. Rice grain consists primarily of starch (~90%) [1], thus, the properties of starch play an important role in determining the ECQ of rice. Rice starch is comprised of two components, amylose and amylopectin, and has many properties such as apparent amylose content (AAC), amylopectin structure, pasting viscosity, gelatinization temperature (GT), gel consistency and texture. All the starch-related properties have various degrees of effect on the ECQ of cooked rice [2].

AAC is widely recognized as the most important factor affecting the ECQ of rice grain [3]. Cooked rice kernels with high AAC (>25%) are dry, separate, less tender, and become hard upon cooling, whereas those with low (12–20%) are glossy, soft, and sticky [4]. Intermediate AAC (20–25%)rice is widely preferred in most rice-producing areas of the world since this kind of cooked rice is soft and flaky [5]. However, AAC alone does not explain all of the variations for ECQ, as cultivars with similar AAC possess different ECQ. The pasting properties of starch, usually measured by Rapid Visco Analyzer (RVA), are also important factors affecting the ECQ of cooked rice. The RVA test simulates the rice cooking process and is a proven tool for the rapid and reproducible assessment of the ECQ of rice. Among these pasting properties measured by RVA, breakdown viscosity (BDV), the difference between peak viscosity (PV) and trough viscosity (TV), is a measure of the ease of disrupting swollen starch granules which indicates the degree of stability during cooking [6]. BDV significantly correlates with gel consistency. Generally, a high BDV indicates a good gel consistency [7]. Setback viscosity (SBV), calculated as the difference between final viscosity (FV) and peak viscosity (PV), exhibits the degree of retro-gradation or hardening of starch upon cooling. A low SBV value is related to softness and to the good sensory qualities of cooked rice. Good ECQ varieties usually have higher BDV and lower SBV and FV. In contrast, the inferior ECQ varieties commonly have lower BDV and higher SBV and FV [8]. This shows that paste viscosity parameters play an important role in evaluating the ECQ of rice.

GT is also closely related to the ECQ of rice [9]. Usually, GT is estimated as the alkali spreading value (ASV) that is assessed by the extent of dispersal of whole milled rice grains in dilute alkali solution (1.7% potassium hydroxide [KOH]) [10]. Rice grains with low, intermediate, and high GT shows complete disintegration, partial disintegration, and no effect in the dilute alkali solution, respectively [10]. Because ASV is measured simply and easily, it has been extensively used to estimate GT in rice quality breeding practices [11]. GT is positively correlated with the amount of time required to cook rice. Rice varieties with high GT require more water and cooking time than those possessing low or intermediate GT. A low or intermediate GT is desired for a high-quality rice variety [12].

The different sensory qualities of cooked rice could meet the requirements of consumers with different cultural and preference background [13].We need to strategize our breeding activities in order to develop and breed systematically rice varieties that meet the expectation of the consumers from the target regions with different taste preferences requirements. An understanding of the relationships among rice AAC, GT, and pasting properties is very important in describing the ECQ of different rice varieties as these have significant implications in rice quality breeding. Although the relationships among the AAC, GT, and pasting properties have been extensively studied [4, 14–18], the correlations were not consistent across these studies, suggesting the existence of complex relationships among them. Most studies focused only on smaller-sized groups of rice varieties or germplasm or recombinant inbred line (RIL) populations derived from bi-parental crosses. Only a few studies had used breeding lines that included both inbreds and hybrids.

Therefore, the present study was undertaken to examine the relationship among AAC, GT, and pasting properties using fixed breeding lines, which could lay the foundation to breed better ECQ rice varieties in a customized manner. The objective of this study was to determine proper ECQ rice cultivars developed in our breeding program for target regions through characterizing the AAC, GT, and pasting properties of their starch. Correlation patterns amongst the ECQ properties were well studied. The chi-square test was used to analyse the independence of different combinations of the categories of AAC and GT. Cluster analysis was then conducted to identify varieties, both hybrids and breeding lines, that possess similar AAC, GT, and RVA parameters matching those of consumer-preferred rice varieties. This research strategy will be useful in the selection of appropriate cultivars that match the consumer-preferred ECQ especially of popular check varieties in the target regions.

## Materials and Methods

### Plant Materials

We collected 671 inbred lines and 116 hybrid rice lines including the famous high-quality rice varieties such as Bg300, Binam, Feng-Ai-Zhan, Huang-Hua-Zhan (HHZ), IR64, Liang-you363, Pei-liang-you1108, and Jin-ke-you651. All the rice materials were planted during the dry season at the International Rice Research Institute (IRRI), Philippines in November 2010. These lines with sufficient grain yields were harvested for the analysis of their physico-chemical properties. After being air dried and stored at room temperature for three months, the samples were milled to white rice using a Satake Rice Machine (Satake Corporation, Hiroshima, Japan) and then ground to pass through a 100-mesh sieve on a Cyclone Sample Mill (UDY Corporation, Fort Collins, Colorado, USA). Finally, the samples were used to identify the AAC, GT, and RVA properties at the Grain Quality and Nutrition Center of IRRI.

### Determination of AAC

The AAC of isolated rice starch was analysed by using the iodine reagent method [19]. Briefly, exactly 25 mg rice flour was gelatinized overnight in 2 ml of 1.0 N NaOH in a water bath set at 50°C. The solution was boiled in the water bath for 10 min and then cooled to room temperature. The cooled solution was extracted three times with 5 ml of butanl:petroleum ether (1:3) to remove the lipid, after which 1.5 ml of 0.4 N KI was added to the solution and mixed. The AC was determined in duplicates with an ART-3 Automatic Titrator according to the manufacturer’s instruction (Hirama Laboratories, Japan) in which 1.57 mM KIO_3_ was titrated at a speed of 2.5 μl per s to the starch solution. The titration terminal was automatically detected with a sensitivity setting of 3, and the used volume of KIO_3_ was transformed into amylose content. Standard amylose solutions were prepared as checks by dissolving pure amylose and amylopectin in distilled water [20].

### Determination of GT

GT was determined using the alkali digestion test [10]. A duplicate set of six whole-milled kernels without cracks was selected and placed in a plastic box (5×5×2.5 cm). Ten mL of 1.7% (0.3035 M) KOH solution was added. The samples were arranged to provide enough space between kernels to allow for spreading. The boxes were covered and incubated for 23 h in a 30°C oven. The starchy endosperm was rated visually based on a seven-point numerical spreading scale as a standard evaluation system for rice [21]. According to the ASV score, GT of rice grains can be classified into four groups: high (1–2), high-intermediate (3), intermediate (4–5) and low (6–7) [11].

### Determination of RVA profiles

The pasting properties of rice flour samples were measured through the RVA (RVA-3D model Thermocline Windows Control and the analysis software, Version 1.2 (New Port Scientific, Sydney, Australia) within a short period of 19 min. About 4 g of flour (12% moisture basis) from each rice sample was weighed directly into the aluminum canister and mixed with about 25 ml of distilled water. The RVA dispersed the samples by rotating the paddle at 960 rpm for the first 10 s of the test, after which the viscosity was sensed using a constant paddle rotation speed of 160 rpm. The idle temperature was set to 50°C and the following 19 min test profiles were run in this order: (1) 50°C held for 1.0 min, (2) temperature was then linearly raised to 93°C until 5 min, (3) temperature was held at 93°C until 12 min, (4) temperature was again linearly lowered to 50°C at 16 min, and (5) temperature was held at 50°C until the full 19 min. All analyses were conducted in triplicates. The pasting parameters PV, TV, FV, BDV, SBV, consistency viscosity (COV = FV—TV), peak time (PKT), and pasting temperature (PT) determined from the RVA curve were recorded. All the viscosity parameters were reported in Rapid Visco Amylograph Units (RVU).

### Statistical analysis

All statistical analyses were performed using the R software [22]. Phenotypic correlations were computed using the “rcorr” function in the R package Hmisc [23]. The chi-square test was calculated using the “chisq.test” function. To find lines with similar ECQ, the whole group was first divided into subgroups according to AAC and GT. Then, within each subgroup, cluster analysis was performed based on the RVA parameters using the “NbClust” function in the “NbClust” package [24] of R. The dissimilarity matrix was computed by “euclidean” method and the cluster analysis method was computed using “ward.D2”.

## Results

### Variation in AAC and RVA pasting profiles

A considerable variation was observed for most of the traits in both inbred group (IG) and hybrid group (HG) (Table 1). For all the traits, a wider range was observed for IG as compared to HG, indicating considerable variations for the observed traits in IG. The mean values of these traits in IG were relatively smaller than those in HG except SBV (53.5 and 51.8 RVU for IG and HG, respectively). The AAC in IG ranged from 9.0% (Cheng-hui448) to 34.1% (KCD1) with a mean of 19.5%, while, the AAC in HG varied from 7.0% (HY290) to 30.8% (IR79118H) with a mean of 20.6%. Among the pasting properties, in IG, BDV was averaged at 101.7 RVU ranging from 0.5 RVU (GSR IR1-15-SU1-Y3-Y1) to 232.2 RVU (GSR IR1-6-D8-Y2), and SBV varied from -123.8 RVU (GSR IR1-6-D8-Y2) to 252.9 RVU (GSR IR1-9-Y5-Y3) with a mean of 53.5 RVU. In HG, BDV was averaged at 107.7 RVU varying from 29.3 RVU (FFZ1) to 177.3 RVU (HanF1-26), and SBV varied from -69.9 RVU (HanF1-26) to 144.7 RVU (FFZ1) with an average of 51.8 RVU (Table 1).

**Table 1:** Descriptive statistics of AAC and RVA properties of 671 inbred and 116 hybrid lines.

Trait^1^	Inbred lines			Hybrid lines		
	Range	Mean±Sd	CV (%)	Range	Mean±Sd	CV (%)
AAC	9.0~34.1	19.5±6.2	32.0	7.0~30.8	20.6±5.3	25.6
PV	121.0~377.6	258.6±41.9	16.2	171.0~353.7	291.5±30.7	10.5
TV	86.2~263.1	157.0±34.4	21.9	114.1~239.3	183.8±19.8	10.8
FV	179.7~500.3	312.1±69.1	22.1	251.1~430.3	343.3±40.9	11.9
BDV	0.5~232.2	101.7±46.8	46.0	29.3~177.3	107.7±28.6	26.6
SBV	-123.8~252.9	53.5±80.9	151.2	-69.9~144.7	51.8±52.2	100.7
COV	82.8~306.8	155.2±42.2	27.2	104.6~209.9	159.5±27.3	17.1
PKT	5.4~6.9	5.8±0.2	3.7	5.6~6.3	5.9±0.1	2.3
PT	65.8~82.2	72.3±3.1	4.2	69.6~79.8	75.4±2.3	3.1

^1^ AAC: Apparent Amylose Content; PV: Peak Viscosity; TV: Trough Viscosity; FV: Final Viscosity; BDV: Breakdown Viscosity; SBV: Setback Viscosity; COV: Consistency Viscosity; PKT: Peak Time; PT: Pasting Temperature; CV: Coefficient of Variation

### Combinations of AAC and GT

**Fig 1:**
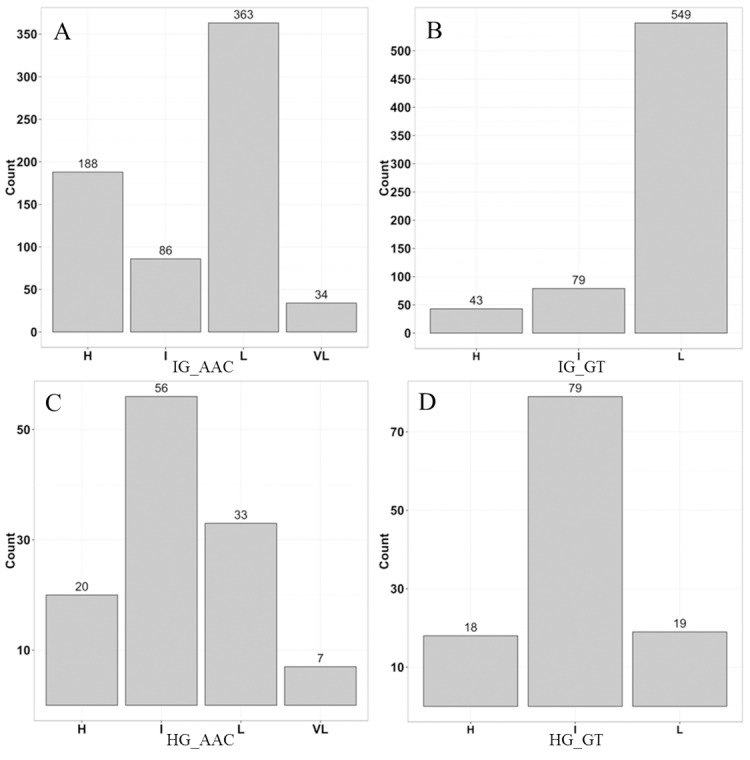
**Histogram distributions of AAC and GT in inbred (A and B) and hybrid groups (C and D)**. H, I, L, and VL were high, intermediate, low, and very low, respectively.

For IG, the AAC ranged from 9.0% to 34.1%, which could be classified into four sub-groups: very low (VL, 9.0–12.0%), low (L, 12.1–20.0%), intermediate (I, 20.1–25.0%), and high (H, 25.1–34.1%). For HG, the AAC ranged from 7.0% to 30.8%, which could also be classified into four sub-groups: VL (7.0–12.0%), L (12.1–20.0%), I (20.1–25.0%), and H (25.1–30.8%). In IG, 50% (336 lines) had low AAC while in HG, 48% (56 lines) had intermediate AAC. A large number of inbred lines (82%) were categorized as having low GT, while in HG, 68% of the lines showed intermediate GT (Fig 1).

Within IG, the VL AAC category comprised of only 34 inbreds, hence, it was combined with the L AAC group. All possible combinations were detected between different AAC and GT categories especially in IG (Table 2). Results of the chi-square test (χ^2^ = 115.7, df = 4, p = 2.2e-16) showed that the combinations of AAC and GT were not random, indicating that AAC and GT were dependent on each other in IG. The observed values for the combinations H / H (AAC / GT) and H / I were lower than the expected values, but the value for H / L was higher than the expected value, indicating that high AAC tended to combine with low GT and was not inclined to combine with either high or intermediate GT. The observed values for the combinations I / H and I / I were larger than the expected values, but the value for I / L was lower than the expected value, indicating that intermediate AAC tended to combine with high or intermediate GT and was not inclined to combine with low GT. The observed value for the combination L / I was lower than the expected value, but the values for L / H and L / L were higher than the expected values, indicating that low AAC tended to combine with high or low GT and was not inclined to combine with intermediate GT (Table 2). Similarly, in HG, the VL AAC group only had seven lines, so it was combined with the L AAC category. All possible combinations between AAC and GT appeared in HG but the chi-square test result (χ^2^ = 9.16, df = 4, p = 0.0573) showed that these combinations were random, indicating that AAC and GT were independent of each other in HG (Table 2).

**Table 2:** The observed and expected values of combinations of different classifications of AAC and GT in inbred and hybrid groups.

		Combinations	GT					
	Pop.		No. of Observed			No. of Expected		
			H	I	L	H	I	L
AAC		H	4	17	167	12	22	153
	Inbred	I	10	38	38	6	10	70
		L	29	24	344	25	47	325
		H	1	15	4	3	14	3
	Hybrid	I	11	41	4	9	38	9
		L	6	23	11	6	27	7

### Relationships between different categories of AAC and RVA pasting profiles

**Fig 2:**
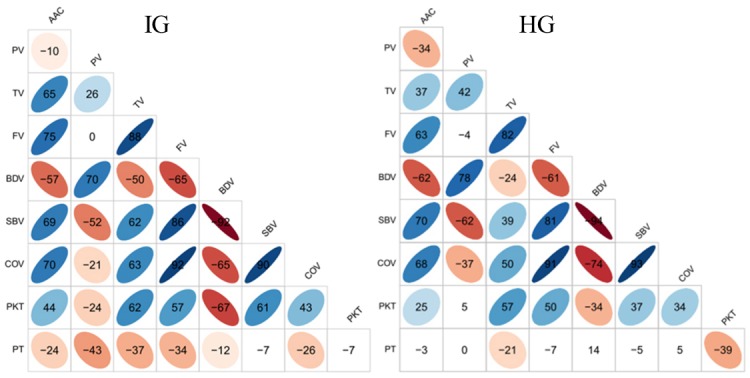
Correlation relationships of AAC and RVA parameters in inbred and hybrid groups. The values were correlation coefficients (r) multiplied by 100. The areas and colors of ellipses showed the absolute value of corresponding r. Right and left oblique ellipses indicated positive and negative correlations, respectively. The values without glyphs indicated insignificant at 0.05.

The relationships of AAC and RVA properties were almost the same for both IG and HG. In both groups, significant positive correlations among AAC, TV, FV, SBV, COV, and PKT were observed with correlation coefficients (r) ranging from 0.43 between COV and PKT to 0.92 between FV and COV in IG and from 0.25 between AAC and PKT to 0.93 between SBV and COV in HG. AAC, TV, FV, SBV, COV, and PKT were all negatively correlated with BDV with r ranging from -0.50 between TV and BDV to -0.92 between SBV and BDV in IG and from -0.24 between TV and BDV to -0.94 between SBV and BDV in HG (Fig 2).

### Phenotypic variance within clusters

Table 3 shows the cluster analysis results based on the AAC, GT, and RVA properties. Overall, IG and HG were separately divided into 52 and 31 clusters distinct to each other in one to three properties of AAC, GT, and RVA. In IG, some clusters were distinct to each other in AAC, GT, and most of the RVA properties simultaneously. For instance, IC4 had higher GT, PV, BDV, and PT but lower AAC, FV, SBV, COV, and PKT than IC36 (Table 3). Some clusters were distinct to each other in two of the AAC, GT, and RVA properties. For instance, IC37 and IC40 had similar AAC but IC37 had low GT, low BDV, and high SBV while IC40 had intermediate GT, high BDV, and low SBV. Both IC7 and IC51 had high GT but IC7 had higher SBV and COV values and lower AAC, PV, TV, FV, and BDV values than IC51. Although the RVA parameters PV, TV, FV, BDV, SBV, COV, and PKT of IC4 and IC25 were similar, IC4 had high GT with AAC ranging from 10.8% to 13.2% and IC25 had low GT with AAC ranging from 17.9% to 18.9% (Table 3). Some clusters were distinct to each other in only one of the AAC, GT, and RVA properties. For instance, IC18 and IC21 had similar AAC and intermediate GT, but IC18 had higher TV, FV, SBV, and COV values and lower PV, BDV, and PT values than IC21. IC5 and IC6 had similar AAC and RVA properties, but GT was intermediate in IC5 and low in IC6. Both IC10 and IC24 had low GT and similar RVA parameters but IC10 had lower AAC than IC24 (Table 3).

**Table 3:** Phenotypic variance within clusters of inbred and hybrid groups.

Cluster^1^	No.	AAC	GT	PV	TV	FV	BDV	SBV	COV	PKT	PT
IC1	3	9.0~10.3	L	218.6~231.6	126.6~139.2	243.8~280.0	88.5~92.4	12.3~53.3	104.7~141.8	5.7~5.9	73.6~76.9
IC2	2	9.5~11.3	I	224.5~254.0	113.0~140.5	250.2~296.8	111.5~113.5	25.7~42.8	137.2~156.3	5.8~6.0	72.9~76.9
IC3	25	10.7~12.3	L	169.8~259.3	86.2~164.3	214.1~295.5	58.6~129.6	-18.4~106.2	110.7~164.8	5.7~6.1	71.3~79.2
IC4	8	10.8~13.2	H	254.3~376.8	135.8~186.0	248.8~318.3	109.1~205.8	-123~12.6	82.8~133.3	5.5~5.8	78.3~81.3
IC5	3	11.1~12.8	I	248.8~294.2	141.9~184.6	260.2~291.8	106.9~131.0	-17.6~11.3	107.3~118.3	5.7~6.2	68.1~74.4
IC6	35	11.4~13.5	L	248.2~340.1	116.7~187.6	216.4~302.7	112.5~180.4	-80.7~16.7	98.2~132.9	5.5~6.1	69.0~73.6
IC7	4	12.0~15.3	H	200.5~237.3	105.4~141.9	243.9~277.9	95.1~104.8	38.8~56.8	134.3~151.8	5.7~5.9	77.5~80.6
IC8	45	12.4~13.4	L	199.3~271.6	106.3~156.6	234.9~311.5	72.8~128.7	-0.3~82.6	120.3~170.0	5.6~6.1	71.2~76.8
IC9	35	13.5~14.2	L	166.6~286.1	90.9~171.3	233.4~384.7	73.1~134.6	-2.8~98.6	122.6~213.3	5.6~5.9	69.6~77.5
IC10	4	13.5~15.6	L	195.5~297.9	179.9~249.0	338.8~441.8	15.6~74.7	77.8~154.9	152.5~192.8	5.9~6.2	68.9~72.8
IC11	40	13.6~14.5	L	243.3~335.8	124.8~182.1	239.3~305.4	95.0~165.3	-46.0~26.6	111.8~149.2	5.5~6.2	70.4~72.9
IC12	27	14.3~15.8	L	183.8~268.5	92.8~171.5	208.0~317.7	71.3~133.7	-16.8~115.3	101.2~187.8	5.7~6.1	70.3~76.0
IC13	6	14.5~16.5	I	217.5~294.8	114.3~208.1	256.6~380.9	36.9~121.3	16.1~148.8	127.8~185.7	5.7~5.9	75.2~77.5
IC14	3	14.6~15.0	H	285.4~317.3	133.0~163.4	244.8~259.8	133.1~179.2	-67.4~-33.3	96.4~111.8	5.6~5.7	78.2~78.2
IC15	31	14.6~15.3	L	264.3~350.0	120.5~167.4	227.3~309.5	104.8~204.5	-93.8~29.8	106.8~149.0	5.5~6.1	68.9~72.9
IC16	24	15.4~16.1	L	265.4~374.8	122.7~194.9	228.8~321.2	110.6~232.2	-123.8~22.0	105.8~144.6	5.5~6.2	68.9~73.7
IC17	8	16.1~17.9	L	219.9~305.0	107.0~193.6	218.7~362.8	54.8~135.8	-24.2~98.6	111.7~184.7	5.4~6.0	69.7~72.9
IC18	2	16.2~17.1	I	272.8~274.2	215.3~232.8	361.9~394.1	41.4~57.5	89.2~119.9	146.7~161.3	6.1~6.3	68.1~71.2
IC19	38	16.2~17.7	L	266.4~367.0	99.8~200.4	210.1~306.2	102.2~228.3	-120.8~4.3	104.7~138.3	5.5~6.2	67.3~76.8
IC20	6	16.3~17.8	H	179.0~243.3	106.9~139.0	248.5~293.0	54.3~117.2	15.7~97.2	132.8~169.8	5.6~6.0	76.8~79.8
IC21	2	16.8~16.9	I	320.8~348.0	189.6~206.3	295.3~319.4	114.5~158.4	-52.7~-1.4	105.8~113.1	5.9~6.3	71.2~72.9
IC22	8	17.2~19.8	I	135.7~256.0	105.0~189.8	250.7~327.8	29.6~124.8	-0.5~115.9	124.3~179.8	5.5~6.0	71.3~79.0
IC23	2	17.4~18.1	H	312.9~329.6	124.2~167.5	237.2~282.5	145.4~205.4	-92.4~-30.4	113.0~115.0	5.4~5.7	69.6~78.3
IC24	4	17.5~20.0	L	237.6~305.9	178.0~242.5	370.8~428.3	10.7~63.4	122.4~153.0	163.7~192.8	5.8~6.4	67.4~74.4
IC25	19	17.9~18.9	L	285.2~377.6	126.2~184.1	230.3~299.8	106.7~218.8	-110.8~9.4	95.3~143.4	5.4~6.2	68.9~71.2
IC26	6	18.4~19.5	H	176.3~233.5	103.8~133.1	265.8~321.3	55.0~102.8	47.8~104.4	138.6~206.3	5.7~5.9	77.5~79.2
IC27	9	19.2~21.9	L	133.8~266.5	95.6~189.3	238.9~377.8	20.8~96.2	69.8~185.1	126.3~225.3	5.7~6.4	69.6~79.2
IC28	5	19.4~20.5	L	275.3~352.9	138.3~166.0	253.4~304.3	112.0~214.7	-99.5~17.1	115.2~143.3	5.5~5.9	68.1~72.1
IC29	3	19.5~20.9	I	274.0~292.9	118.3~137.3	254.6~279.5	155.7~156.5	-25.5~-13.2	131.0~142.5	5.5~5.7	73.5~75.2
IC30	20	20.5~22.6	I	163.5~234.8	113.8~177.9	246.0~304.3	39.7~110.8	33.3~114.0	119.6~172.5	5.6~6.5	75.9~79.8
IC31	8	20.5~23.0	H	222.3~258.1	121.1~149.2	256.7~293.9	73.2~118.7	18.8~71.6	127.3~144.8	5.6~5.9	76.0~78.2
IC32	3	21.4~22.5	L	291.8~331.3	140.3~166.8	256.8~293.4	125.8~189.3	-74.5~1.7	90.0~138.8	5.5~5.9	68.2~72.8
IC33	6	21.8~23.3	L	217.3~275.2	151.7~182.5	362.0~413.9	56.5~92.7	97.3~167.3	190.0~257.6	5.8~6.0	70.4~72.8
IC34	12	22.2~23.8	L	182.7~297.3	119.3~167.5	238.5~334.4	53.8~129.8	37.1~117.5	119.3~189.3	5.6~6.1	68.1~75.2
IC35	13	22.8~24.1	I	177.8~245.8	120.8~161.5	247.1~332.5	16.3~104.2	30.3~154.8	123.6~171.0	5.7~6.1	76.0~77.8
IC36	18	23.9~26.0	L	198.3~265.3	163.3~212.8	359.3~496.8	22.8~82.3	108.3~252.9	156.6~306.8	5.9~6.3	69.7~76.8
IC37	7	24.1~25.4	L	168.2~290.8	126.2~171.1	270.8~384.9	12.2~119.8	30.7~216.8	144.6~228.9	5.6~6.1	67.3~79.0
IC38	12	24.4~27.2	I	132.2~259.8	90.8~195.0	197.9~404.4	22.2~122.3	49.5~195.3	107.2~222.8	5.5~6.3	74.4~78.3
IC39	2	24.5~26.8	H	205.1~223.7	144.6~145.3	289.8~315.0	60.5~78.3	84.7~91.3	145.2~169.7	5.9~5.9	76.9~78.4
IC40	2	24.8~25.3	I	261.2~280.8	125.8~133.0	276.4~292.8	135.4~147.8	-4.3~31.6	143.4~167.0	5.5~5.5	72.9~76.8
IC41	1	25.0	H	284.3	120.0	251.6	164.3	-32.8	131.6	5.5	75.3
IC42	2	25.1~26.8	L	308.8~355.6	131.0~166.3	262.5~286.8	177.8~189.3	-68.8~-46.3	120.5~131.5	5.5~5.8	69.6~69.7
IC43	54	26.3~27.7	L	202.4~300.0	163.1~254.8	341.5~481.8	16.1~81.1	115.5~232.3	138.8~291.3	5.9~6.5	67.4~74.4
IC44	6	26.5~28.0	L	128.5~234.9	113.3~164.0	211.8~362.9	0.5~106.4	63.4~170.2	83.8~212.8	5.7~6.9	65.8~82.2
IC45	46	27.9~29.0	L	208.0~326.6	190.8~246.9	357.8~500.3	10.1~134.3	79.9~236.2	166.9~277.7	5.7~6.3	66.6~72.8
IC46	4	28.0~29.5	I	203.9~295.1	182.8~227.4	326.9~404.4	21.2~101.6	92.1~123.0	144.2~210.9	5.7~6.3	75.2~77.5
IC47	19	29.1~29.9	L	209.3~314.8	170.7~263.1	358.6~455.7	16.7~104.6	99.2~178.3	147.3~247.3	5.7~6.5	66.6~72.0
IC48	4	29.2~31.6	L	121.0~290.8	95.3~178.1	179.7~379.0	25.8~132.4	32.1~115.9	84.4~200.9	5.7~5.8	67.3~72.9
IC49	2	29.5~31.8	L	321.6~359.3	183.4~183.9	291.9~296.1	137.7~175.8	-67.3~-25.5	108.5~112.2	5.8~6.1	71.2~71.3
IC50	18	30.0~31.3	L	214.1~316.5	183.3~242.5	357.9~460.3	18.1~104.8	123.2~171.3	163.1~255.8	5.5~6.2	66.6~72.0
IC51	3	30.8~34.1	H	276.4~299.8	156.35~178.4	283.4~293.3	100.8~130.2	-11.6~14.1	114.9~127.0	5.7~5.7	78.65~79.8
IC52	2	30.8~32.3	I	272.2~295.8	169.6~203.2	313.4~353.8	92.7~102.6	41.3~58.0	143.8~150.7	5.8~6.1	72.8~78.3
HC1	1	7.0	L	320.7	164.1	285.0	156.6	-35.7	120.9	6.0	71.3
HC2	1	8.2	I	201.9	134.9	288.8	67.0	86.9	153.9	5.7	75.2
HC3	5	10.1~13.2	I	278.4~300.8	178.0~198.5	338.3~381.5	91.3~109.2	51.2~85.3	160.3~183	5.7~5.9	76.0~77.6
HC4	4	10.6~13.2	I	309.0~332.2	164.9~177.6	272.9~287.2	143.4~154.6	-45~-35.6	107.8~111.6	5.7~5.8	77.5~78.3
HC5	4	11.4~15.7	H	290.7~353.7	153.3~176.4	276.8~290.6	134.3~177.3	-69.9~-13.8	107.3~123.5	5.7~5.8	76.8~79.8
HC6	6	12.7~14.7	L	294.1~328.8	157.8~174.9	281.2~296.3	120.6~153.8	-32.5~0.8	113.2~126.7	5.7~6.1	71.2~73.7
HC7	4	12.7~15.4	I	280.3~329.2	167.6~188.0	296.1~307.3	112.7~151.7	-32.3~17.5	119.3~130.2	5.9~6.1	72.0~72.9
HC8	5	13.9~14.8	I	309.5~335.5	165.6~171.0	275.4~288.3	141~164.5	-59.9~-23.3	104.6~119.8	5.7~5.9	75.9~77.6
HC9	1	15.0	L	343.1	172.6	284.8	170.5	-58.3	112.3	5.7	77.6
HC10	1	15.3	H	347.3	196.1	332.3	151.3	-15.0	136.3	6.1	71.3
HC11	2	15.5~15.8	I	282.1~292.0	166.8~191.4	313.3~361.3	100.6~115.3	31.3~69.3	146.5~169.9	5.8~5.9	76.0~77.6
HC12	2	17.0~17.2	L	317.7~325.8	166.0~179.8	281.1~311.4	145.9~151.7	-36.6~-14.3	115.1~131.6	5.9~5.9	71.4~72.1
HC13	1	18.0	I	291.8	177.5	321.1	114.3	29.3	143.6	6.0	71.3
HC14	8	19.5~23.2	H	284.6~316	185.2~202.1	353.2~382.8	94.7~126.6	40.9~85.8	166.9~188.4	5.6~6.0	76.0~77.6
HC15	1	19.5	L	250.1	220.8	394.8	29.3	144.7	173.9	6.0	69.6
HC16	2	20.0~20.1	I	299.7~316.8	189.3~196.8	343.1~372.3	102.9~127.5	26.3~72.7	153.8~175.6	5.9~5.9	75.9~75.9
HC17	2	20.4~22.6	H	248.7~256.8	174.9~178.1	336.8~358.3	70.6~81.8	80.1~109.6	161.9~180.2	5.9~5.9	76.0~78.2
HC18	12	21.5~22.8	I	260.3~310.3	177.3~205.8	359.8~382.0	74.3~113.4	54.0~120.6	167.4~194.8	5.8~6.1	74.4~78.3
HC19	1	21.6	L	308.1	184.5	333.3	123.6	25.3	148.8	5.9	71.2
HC20	3	22.2~24.0	L	293.6~302.8	203.3~215.8	377.6~402.2	79.7~93.5	80.8~99.4	167.8~186.4	6.0~6.1	70.4~71.3
HC21	8	22.6~24.5	I	195.6~266.0	124.2~181.4	274.7~353.3	71.4~95.9	59.3~110.5	150.5~185.2	5.7~5.9	75.1~76.9
HC22	18	23.1~24.1	I	266.4~305.0	161.6~201.8	342.3~392.4	64.6~125	47.6~120.0	158.4~209.9	5.8~6.2	73.6~77.6
HC23	1	23.8	I	288.3	165.3	287.0	123.1	-1.3	121.8	5.7	79.1
HC24	1	23.9	I	323.5	178.9	311.8	144.6	-11.7	132.9	6.0	76.0
HC25	3	23.9~25.2	H	277.0~306	174.0~185.8	347.5~371.9	103~121	41.5~72.3	162.5~186.1	5.7~5.8	75.1~76.8
HC26	6	25.1~26.5	I	276.2~308.8	177.5~197.1	334.7~381.0	92.3~112.3	51.2~104.8	154.2~197.2	5.7~6.0	74.4~77.6
HC27	2	25.5~26.8	I	205.3~251.9	155.8~175.1	343.4~359.7	49.5~76.8	107.8~138.1	184.6~187.6	5.9~5.9	74.5~76.0
HC28	2	25.6~26.2	L	294.9~305.1	203.4~218.6	383.9~413.7	86.5~91.5	89.0~108.6	180.5~195.1	5.8~6.0	69.6~71.3
HC29	5	26.9~29.0	I	300.3~332.1	203.8~236.9	372.3~430.3	88.0~105.8	70.9~105.3	168.5~193.3	5.9~5.9	74.3~76.7
HC30	2	28.2~29.6	L	241.7~299.3	203.3~239.3	365.9~416.3	38.3~60.0	117.1~124.3	162.6~177.1	6.2~6.3	70.5~70.5
HC31	2	28.6~30.8	I	171.0~233.6	114.1~178.7	251.1~375.3	54.9~56.9	80.1~141.8	137.0~196.7	5.6~5.9	72.1~75.2

^1^ IC and HC indicated the clusters of inbred and hybrid groups, respectively.

Similar results were found in HG. Some clusters were distinct to each other in terms of AAC, GT, and most of the RVA properties simultaneously. For instance, HC6 had higher PV and BDV but lower AAC, GT, SBV, COV, and PT than HC21 (Table 3). Some clusters were distinct to each other in two of the AAC, GT, and RVA properties. For instance, AAC was similar between HC20 and HC21 but GT was low in HC20 and intermediate in HC21. HC20 also had higher PV, TV, and FV but lower PT than HC21. Both HC5 and HC14 had high GT, but the BDV of HC5 was higher than that of HC14 and the AAC, TV, FV, SBV, and COV values of HC5 were lower than those of HC14. Although the RVA parameters in HC14 were similar to those in HC26, the AAC of HC14 was lower than that of HC26 while GT was high in HC14 but low in HC26 (Table 3). Some clusters were distinct to each other in just one of the AAC, GT, and RVA properties. HC3 and HC4 had similar AAC and intermediate GT, but HC3 had higher TV, FV, SBV, and COV and lower PV and BDV than HC4. The AAC and RVA properties were similar between HC6 and HC7, but the GT was low in HC6 and intermediate in HC7. Both HC3 and HC18 had intermediate GT and similar RVA parameters, but the AAC in HC3 was significantly lower than that in HC18. Therefore, the results showed that the clusters were distinct to one another in at least one of the AAC, GT, and RVA parameters. Also, cluster analysis was an effective way to distinguish lines with different AAC, GT, and/or RVA parameters and to classify lines with similar AAC, GT, and RVA parameters into the same cluster.

## Discussion

### Combinations of AAC and GT

All possible combinations of AAC and GT were observed in both IG and HG but the chi-square test showed that these combinations were random in HG but non-random in IG. In the study conducted by Hossaina et al. [5] on the cooking and eating characteristics of 17 newly-identified inter-sub-specific (*indica / japonica*) rice hybrids, not all the possible combinations of AAC and GT appeared. Nonetheless, we performed the chi-square test using their data and found that the difference between the observed value and expected value was not significant (χ^2^ = 5.65, df = 4, p = 0.2272). These results indicate that AAC and GT were independent of each other and their combinations were random in the hybrid lines studied by Hossaina et al. [5]. The absence of some possible combinations was just attributed to their low probability and smaller group size.

Some studies also found the non-random combinations of AAC and GT in worldwide collected inbred accessions [12]. They found that high AAC rice usually had intermediate or low GT while low AAC rice usually had high or low GT, which made it difficult to find the combinations of high AAC with high GT or low AAC with intermediate GT. Similarly, Yang et al. [25] reported the non-random combinations of AAC and GT in 379 non-waxy rice accessions and found that high AAC rice accessions always had intermediate and low GT and low AAC accessions always had high and low GT. Kong et al. [26] also reported the non-random combinations of AAC and GT in 14 rice cultivars produced in China and concluded that waxy rice and low AAC rice cultivars had both high and low GT classes; intermediate AAC rice cultivars only had intermediate GT class; and high AAC rice cultivars had both intermediate and low GT classes. The non-random combinations of AAC and GT were also observed in waxy rice accessions [27]. In the present study, all possible combinations of AAC and GT appeared in IG, but the chi-square test showed that these combinations were not random (χ^2^ = 115.7, df = 4, p = 2.2e-16). The observed values for the AAC / GT combinations H / L, I / H, I / I, L / H, and L / L were significantly higher than the expected values whereas, in contrast, the combinations H / H, H / I, I / L, and L / I had significantly lower values than the expected values. Therefore, we could conclude that all possible AAC / GT combinations may appear in both hybrid and inbred lines as long as the group size is big enough, but the combinations were non-random in the latter group. For inbred lines, high AAC tended to combine with low GT, intermediate AAC tended to have high or intermediate GT, and low AAC tended to have high or low GT.

Therefore, the genetic relationship between AAC and GT may be different for hybrid lines and inbred lines. Tian et al. [3] carried out a candidate-gene association mapping study for the ECQ of rice using 70 rice varieties (33 *indica* and 37 *japonica*) and found that *Wx* functioned as the sole major gene for AAC and as a minor gene affecting GT, whereas *SSII-3* was the sole major gene controlling GT and was a minor gene affecting AAC [3]. Therefore, for inbred lines, both *Wx* and *SSII-3* genes might affect AAC and GT simultaneously which could account for the non-random combinations of AAC and GT. However, for hybrid lines, *Wx* might just had an effect on AAC only but not on GT and *SSII-3* only had an effect on GT but not on AAC, so that AAC and GT were independent of each other and their combinations were random. However, the hybrids are products of the F_2_ segregating generation, which is tested for grain quality and is dependent on the parental range for each hybrid. This, therefore, supports AAC and GT to be independent of each other and their combinations to be random in hybrid lines.

### Complex correlations

Correlation analysis showed positive or negative relationships between AAC and RVA parameters. Correlations between these parameters have been extensively explored by previous studies with only some correlations consistent across different studies. Consistent positive correlations among AAC, SBV, and COV were observed in different studies; PKT was always positively correlated with FV, SBV, and COV; and TV and FV were always positively correlated with each other. Also, BDV and SBV were found to be negatively correlated with each other across previous studies [4, 14–18, 28]. These correlations were in agreement with the results obtained in this present study. These stable correlations may be the results of the physicochemical relationships between these traits which breeders can utilize for selection in advanced breeding generations [29]. Some inconsistent correlations may be highly influenced by the size of the group studied. In this study, strong positive correlations between FV and SBV (0.86 in IG and 0.81 in HG) and between FV and COV (0.92 in IG and 0.91 in HG) were observed, which were consistent with findings of most of the previous studies [4, 14–18] but were not significant in the study by Sun et al. [28]. In that study, the correlation coefficients were 0.65 (FV and SBV) and 0.59 (FV and COV), but only eight rice varieties were studied, thereby rendering these correlations insignificant. To get credible correlations, proper group size is, therefore, necessary.

Another possible reason affecting the inconsistency of correlations between AAC and RVA parameters may be the level of AAC. In the present study, AAC ranged from 9.0% to 34.1% in IG and showed a positive correlation (r = 0.65) between AAC and TV, which was in agreement with the findings of Wang et al. [17] where the range of AAC as 9.2%–31.5% and a positive correlation of 0.82 were observed. However, in another study by Chen et al. [14], AAC was in the range of 10.3%–25.4% without high AAC category and a negative correlation (r = -0.75) was reported between AAC and TV. This illustrates that the level of AAC may affect the relationship between AAC and TV.

Besides no apparent physicochemical relationship between these traits that may contribute to the inconsistency of correlations. For instance, in the present study, no significant correlation between PT and SBV was observed in both IG (-0.07) and HG (-0.05), which was consistent with the findings of Xu et al. [18] where r was 0.10. Although a significant positive correlation between PT and SBV was observed in the studies by Wang et al. [17] and Hsu et al. [15], r was very small at only 0.18 and 0.23, respectively. The weak correlation between PT and SBV indicates that maybe no apparent physicochemical relationship exists between them, so that the correlation coefficient between them was inconsistent across different studies.

### Customized breeding of rice cultivars for different regions

The ideal way for breeders to derive good quality varieties that will cater to the varied interests of consumers across rice-consuming countries is by screening the breeding materials for ECQ and keeping the popularly preferred quality varieties in their study [30]. Cluster analysis is an effective way to classify lines with similar AAC, GT, and RVA parameters into the same cluster. By using this strategy, we found some rice cultivars having AAC, GT, and RVA properties that were similar to those of popular high-quality rice varieties. For instance, Huang-Hua-Zhan (HHZ) is a high-quality inbred rice variety from China which has a low AAC (14.2%), low GT, high BDV (128.2 RVU), and low SBV (16.8 RVU). Through cluster analysis, we found that 34 inbred cultivars in IC9 had similar AAC, GT and RVA parameters to those of HHZ, so the ECQ of these lines may be similar to that of HHZ (Table 3). In addition, these lines had higher grain yield and possessed tolerance to abiotic stresses such as drought, salinity, and submergence conditions. These newly-developed high-yielding lines could, therefore, be planted in highly-adaptable HHZ varietal areas where consumer preference is already established along with the variety’s superior performance under drought, saline, or submergence stress conditions. Interestingly, HHZ was recently found to be adaptable across 16 locations in eight countries and its grain quality was acceptable. In China alone, it occupies a cumulative cultivation area of six million hectares. New breeding materials with similar ECQ as that of HHZ and have additional abiotic stress tolerance traits could, thus, be introduced to increase genetic diversity and, thereby, reduce genetic vulnerability. IR64 is a high-quality inbred line developed by IRRI which has an intermediate AAC (21.2%) and was classified into IC30, indicating that the lines in IC30 had similar AAC, GT, and RVA properties as that of IR64 (Table 3). Fourteen of these lines had improved grain yield, drought tolerance, salinity tolerance, or bacterial blight tolerance and had similar ECQ as IR64. IR64, which has been widely adopted across South and South East Asia because of its superior grain quality and grain yield, could now be replaced with superior high-yielding, multiple stress-tolerant cultivars which have the same ECQ as IR64. Amongst the 14 lines in the IC30 cluster, the lines IR83140-B-11-B and IR83140-B-36-B, developed by our group and which have already been released in the Philippines as GSR 11 and NSIC Rc29, respectively, showed a similar ECQ as that of IR64. Binam is a highly preferred and popular Iranian aromatic variety with excellent ECQ, an AAC of 18.4%, and high GT. We found five lines with improved grain yield and tolerance to drought and salinity in cluster IC26 that had similar AAC, GT, and RVA parameters to that of Binam (Table 3), which could provide farmers with higher grain yield under drought or salinity conditions in Iran. However, the intensity of their aroma needs to be matched for them to be successful and to be widely adopted. Bg300 is a high AAC (25.2%) and intermediate GT rice variety, which is popular with consumers in Sri Lanka because of its hardy texture when cooked. Interestingly, we found that IR72 (25.1% AAC and intermediate GT) developed by IRRI, BR11 (24.4% AAC and intermediate GT) from Bangladesh, and two of our breeding lines GSR IR1-5-Y3-S2-SU1 (25.5% AAC and intermediate GT) and GSR IR1-5-Y3-S4 (24.8% AAC and intermediate GT) in cluster IC26 had similar AAC, GT, and RVA properties to those of Bg300 (Table 3). However, the Sri Lankan market preference is leaning towards short bold grains over medium slender or long slender grains. Thus, it is important to match the grain shape and size of a variety with the prevailing preference especially for Sri Lanka to successfully replace Bg300 despite its grain yield performance under adaptation trials. Feng-Ai-Zhan (FAZ) is a good ECQ rice variety from China with 20% AAC and low GT which had shared the same IC27 cluster with our newly-developed cultivars that possess improved grain yield and multiple abiotic stress tolerance. This indicates that FAZ and these cultivars have similar AAC, GT, and RVA properties. Thus, these newly developed lines once tested for their grain yield performance and adaptation in FAZ areas could potentially replace this variety.

For hybrid lines, the popular two-line hybrid rice variety Liang-you363 from China has relatively better ECQ (17.0% AAC and low GT). One of the drought-tolerant hybrid lines i.e. HanF1-35 in the HC12 cluster clade had similar AAC (17.2%), GT (low), and RVA parameters, indicating a comparable ECQ with that of Liang-you363 (Table 3). Likewise, Pei-liang-you1108 is a relatively good ECQ (24.5% AAC and intermediate GT) two-line hybrid variety from China and we found seven lines in the HC21 cluster clade that had similar AAC, GT and RVA properties to those of Pei-liang-you1108, hence with comparable ECQ (Table 3). Jin-ke-you651 is another relatively better ECQ two-line hybrid rice variety from China which had similar AAC, GT, and RVA properties to those of 11 hybrid lines within the HC18 cluster clade, thereby indicating that they have the same ECQ (Table 3).

Further studies need to be carried out for the evaluation of these promising lines with an ECQ that is comparable to that of the elite and consumer-preferred varieties under the target environments. These improved cultivars with better ECQ that were developed through this novel approach in our study helped to identify several customized varieties with improved grain yield and multiple abiotic stress tolerances against salinity, drought, and/or submergence conditions for different target regions. These lines have high ECQ in normal conditions and improved grain yield in both normal and abiotic stress conditions, but it remains to be seen whether these lines could have high ECQ in abiotic stress conditions. Further research in this direction is, therefore, required.

## Abbreviations

AAC: Appearance amylose content
BDV: Breakdown viscosity
COV: Consistency viscosity
CV: Coefficient of Variation
ECQ: Eating and cooking quality
FV: Final viscosity
GC: Gelatinization temperature
HG: Hybrid group
IG: Inbred group
PKT: Peak time
PT: Pasting temperature
PV: Peak viscosity
RVA: Rapid visco analyzer
RVU: Rapid Visco Amylograph Units
SBV: Setback viscosity
TV: Trough viscosity
